# Health literacy and associated factors among adult type 2 diabetic patients in Woldia Comprehensive Specialized Hospital, North-East Ethiopia, 2022

**DOI:** 10.3389/fpubh.2025.1502852

**Published:** 2025-02-18

**Authors:** Habtemariam Mulugeta Abate, Prem Kumar, Samuel Anteneah, Mitaw Girma, Wondosen Yimam, Birhanu Desu

**Affiliations:** ^1^Department of Biomedical Sciences, College of Medicine and Health Sciences, Wollo University, Dessie, Ethiopia; ^2^Department of Surgical Nursing, College of Medicine and Health Sciences, Wollo University, Dessie, Ethiopia; ^3^Department of Comprehensive Nursing, College of Medicine and Health Sciences, Wollo University, Dessie, Ethiopia; ^4^Department of Emergency Medicine and Critical Care Nursing, Wollo University, Dessie, Ethiopia

**Keywords:** diabetes, Ethiopia, health literacy, magnitude, Woldia

## Abstract

**Background:**

Despite limited research, health literacy in developing countries like Ethiopia remains low, making it a significant challenge to combat non-communicable diseases.

**Objective:**

To assess the level of health literacy and associated factors among adult type 2 diabetic patients in Woldia Comprehensive Specialized Hospital, North-East Ethiopia, 2022.

**Methods and materials:**

A cross-sectional study was conducted at Woldia Comprehensive Specialized Hospital from April 19 to July 19, 2022, involving 423 participants. Systematic random sampling, face-to-face interviews, and document reviews were used to collect data. The data were coded and entered into Epi-Data version 4.6.0.2, then analyzed using Statistical Package for the Social Sciences (SPSS) version 26. The results were presented in texts, tables, and figures. Bivariable and multivariable logistic regression analyses were performed to identify significant predictors with *p*-values below 0.05 and to verify the assumptions of binary logistic regression.

**Result:**

General Health Literacy Index was 27.86 (±6.71), and the magnitude of participants with adequate health literacy in the study was 22.2% (95% CI: 18.4–26.3%). Ages (18–35) years [(AOR =14, 95% CI = 3.86–50.77)], ages (36–50) years [(AOR = 15.38, 95% CI = 4.23–55.9)], being male [(AOR = 2.945, 95% CI = 1.570–5.526)], no experiencing depression symptoms [(AOR = 2.673, 95% CI = 1.308–5.463)], not experiencing anxiety [(AOR = 2.001, 95% CI = 1.011–3.960)] and being literate [(AOR = 4.120, 95% CI = 1.397–12.146)] were significantly associated with adequate health Literacy.

**Conclusion and recommendations:**

The magnitude of adequate health literacy was low. Ages (18–35) years, ages (36–50) years, being male, not experiencing depression symptoms, not experiencing anxiety, and being literate were significantly associated with adequate health literacy. Health professionals should regularly implement health education programs for diabetic patients and communities, focusing on females, older adults, the illiterate, and those experiencing depression and anxiety.

## Introduction

Metabolic problems are the hallmark of diabetes mellitus, type I diabetes, which is more frequent in young people, and type II diabetes, which is more common in adults, are the two main forms of diabetes (1). Low-to-medium-wage countries have low education levels, exacerbating health literacy (HL) effects. In 2020, the WHO reported that deaths due to diabetes mellitus in Ethiopia numbered ~16,258, accounting for about 2.88% of total deaths. The adjusted death rate was 33.58 per 100,000 people, ranking Ethiopia 80th in the world and 7th in the country among other diseases (2, 3). Currently, diabetic micro- and macro-complications are increasing rapidly in Ethiopia due to poor glycemic control (3).

Health literacy empowers patients to make informed decisions, actively engage with healthcare providers, and understand disease prevention strategies, including lifestyle modifications, medication adherence, and self-monitoring (4). This enhanced understanding ultimately improves self-management and helps control complications associated with diabetes (4). The quality of life for patients with type 2 diabetes can be significantly improved through a combination of reading skills, knowledge, self-care behaviors, decision-making, communication, and access to health information (5, 6). Three studies conducted in Iran strongly support this conclusion by demonstrating the importance of health literacy promotion programs in helping individuals with the disease manage their health more effectively (5–7). However, empowering communities with health literacy remains a significant challenge throughout the world (8–11). Especially Research conducted in Ethiopia on health-related quality of life among diabetic patients has shown the mean scores for the physical health, social health, psychological health, and environmental health domains are all below 50 (12–14). Additionally, the occurrence of complications such as retinopathy, nephropathy, neuropathy, stroke, heart attack, erectile dysfunction, diabetic foot ulcers, diabetic ketoacidosis, hypoglycemia, and other issues is dramatically increasing (15–23). This underscores the need for researchers to prioritize assessing the health literacy status of the community. The probability of dying after being admitted to the hospital for chronic conditions has been associated with lower health literacy (24, 25). The decline in the expansion of health literacy among the general population negatively impacts individuals' psychological, social, and cultural wellbeing. It also leads to increased emergency room visits, which results in higher costs and greater resource use for health institutions, while affecting personal and family life (26–31).

Some studies have indicated that drugstore expenses were higher among respondents with low health literacy compared to those with high health literacy, ranging from $12 to $1,110 (32). Low health literacy skills contribute to an annual increase in healthcare spending of $73 billion, whereas improving health literacy skills could save over $25 billion annually by preventing 1 million hospital visits (26–32). According to research conducted in various countries, the key factors impacting health literacy include age, gender, marital status, educational attainment, ethnicity, and lack of awareness about diabetes (17, 32–37).

According to the Ethiopian National Healthcare Quality Strategy plan Ethiopian government working on the establishment of health education units, peer learning to improve patient care, and the prediction and discussion of patient health needs (38). Despite these efforts, the level of health literacy regarding type 2 diabetes in the community remains a significant challenge.

Currently, the incidence and complications of type 2 diabetes are rapidly increasing worldwide, particularly in Ethiopia. In contrast, various scholars have primarily focused on medical approaches while overlooking the impact of health literacy on diabetic self-care behaviors. Additionally, factors such as membership in diabetic associations, personal glucometer use, distance from health institutions, and khat consumption have not been studied by previous researchers in Ethiopia. This lack of research on health literacy related to type 2 diabetes in Ethiopia is another significant issue. Consequently, ongoing research on health literacy concerning type 2 diabetes is crucial for addressing the incidence, morbidity, mortality, and complications associated with this condition. Therefore, the main goal of this study is to fill the gaps in understanding health literacy among type 2 diabetes patients by introducing important new variables that have not been previously addressed by scholars in Ethiopia. So, this study aimed to assess the magnitude of health literacy and associated factors among adult Type 2 diabetic patients receiving follow-up care at Woldia Comprehensive Specialized Hospital from April 19 to July 19, 2022.

## Method and materials

### Study setting and period

The study was conducted at Woldia Comprehensive Specialized Hospital, one of the governmental hospitals at the zonal level located in northeastern Ethiopia. The research took place from April 19 to July 19, 2022. This hospital is a vital healthcare institution serving a population of ~1.5 million people. It offers a wide range of medical services, including medical, surgical, obstetric, and orthopedic care. The hospital employs 406 healthcare workers and 240 administrative staff, ensuring comprehensive service delivery. Among its specialized services is a diabetes follow-up program that caters to 1,150 patients with Type 2 diabetes mellitus (T2DM). During the study period, ~1,000 diabetic patients attended follow-up appointments scheduled on various dates.

Study Design: An institutional-based cross-sectional study design was employed to evaluate the targeted population. The study focused exclusively on adult patients with Type 2 diabetes who were receiving follow-up care at Woldia Comprehensive Specialized Hospital. Participant's eligibility criteria: The study focused on adult patients with Type 2 diabetes who had consistently attended follow-up care at least 6 months prior to the study. However, patients who were unable to communicate due to severe medical conditions during the data collection period were excluded. Participants were systematically selected to ensure a diverse and representative sample of adult patients with Type 2 diabetes.

Study Variables: The dependent variable in this study is adequate health literacy, which reflects an individual's ability to effectively understand and utilize health-related information. Several independent variables influence this outcome, which can be categorized into socio-demographic, behavioral, psychosocial, and clinical factors. Socio-demographic factors include age, average monthly income, occupational status, educational attainment, gender, marital status, and enrollment in community-based health insurance, residency and distance from health institutions collectively influence an individual's access to resources and opportunities for health education. Additionally, Behavioral and psychosocial factors—such as alcohol consumption, khat use, depression, anxiety, and stress—significantly impact health literacy by affecting both the cognitive and emotional capacities to process health information. Additionally, clinical factors such as chronic complications of diabetes mellitus, the number of chronic complications, the duration of diabetes mellitus, treatment regimens, comorbid conditions, self-rated health, membership in a diabetic association, ownership of a personal glucometer, and knowledge of diabetes management play a significant role.

Operational Definitions: Adequate Literacy: if the study participants scored from the General Health Literacy 47-item index questionnaire > 33 points (33, 39–41). Limited Health Literacy: if the study participants scored from the General Health Literacy 47-items index questionnaire ≤ 33 points (33, 39–41). Diabetic Knowledge: study participants with a score greater than or equal to 80% from 14–items in the diabetic knowledge questionnaire had adequate knowledge, while the patients with scores below 80% had inadequate knowledge (29). Self-Care: study participants who scored equal to or greater than the mean score were classified as having good diabetes self-care practice and those who scored below the mean were considered to have poor self-care practice (42).

Self-Rated Health: In this study, it is defined as “good” when the patient reported having excellent, very good, or good health and “poor” when the Participant reported having fair or poor health, according to the Stanford Patient Education Research Center Self-Rated Heath Scale (42). Co-morbidity is an illness or disorder that coexists with diabetes but is mainly unrelated (33). Depression: study participants were scored, Normal: 0–9, Depressed ≥10 (15, 16). Anxiety: study participants were scored, normal: 0–7, Anxious: ≥8 (15). Stress: study participants were scored, normal: 0–14, Stressed: ≥15 (15). Alcohol Drinker: It is defined as the proportion of individuals who have ever used alcoholic drinks such as “tela, tej, katicala/areke, beer, wine, or other drinks that can cause intoxication at least once” in his/her lifetime or respondent who drank alcohol during 1 month preceding the study at least once per month (17, 18). Khat chewing: respondent who chewed the leaves of the khat plant during his lifetime in any amount (17, 18).

### Sample size determination and sampling technique

The study sample size was calculated by assuming the prevalence of adequate health literacy at 50% since there was no adequate research done in Ethiopia. The sample size was calculated using the following formula with 95% CI, *d* = 0.05, *Z* = 1.96, *P* = 50%

${\text{n}}_{\text{i}}\text{=}\frac{{\text{z}}^{\text{2}}\text{p}\text{.}\text{q}}{{\text{d}}^{\text{2}}}$ ${\text{n}}_{\text{i}}\text{=}\frac{{\text{z}}^{\text{2}}\text{p}\text{.}\text{q}}{{\text{d}}^{\text{2}}}\text{=}\frac{{\text{(}\text{1}\text{.}\text{96}\text{)}}^{\text{2}}\left(\text{0}\text{.}\text{5}\right)\left(\text{0}\text{.}\text{5}\right)}{{\text{(}\text{0}\text{.}\text{05}\text{)}}^{\text{2}}}\text{=}\frac{\text{0}\text{.}\text{9604}}{\text{0}\text{.}\text{0025}}\text{=}\text{384}$, after that adding a 10% non-response rate =38.4 + 384 = 422.4 = 423, *n* = 423.

The response rate was calculated based on the rates reported in previous studies conducted in similar health settings. In earlier research conducted in Ethiopia, the response rate was 90%. For this study, the non-response rate was estimated at 10%. After analyzing our data, the response rate was determined by dividing the total number of participants who provided complete information during data collection by the total sample size, and then multiplying by 100 to express it as a percentage. To contact potential participants, the following steps were followed : First, ethical clearance was obtained from Wollo University's Ethical Review Committee, along with administrative permission from the CEO of Woldia Comprehensive Specialized Hospital. After receiving permission from the CEO, a systematic random sampling method was employed during data collection. The value was calculated as follows: First, the total number of type two diabetic patients who had treatment follow-ups on different appointment dates within the study period was determined by counting the appointment dates listed in the registration book from the previous year. The value was calculated as *K* = 1,000/423 = 2, where *N* = 1,000 and *n* = 423. Subsequently, the first participant was selected using a random lottery method. The next participant was then selected at every two-interval until the required sample size was achieved.

### Data collection tools and procedures

Socio-demographic factors: were developed from the literature review and conceptual framework. Health Literacy: The second half of the survey is based on the General Health Literacy Index from the HLS-EU-Q47. This index covers three domains: disease prevention, health promotion, and healthcare. The survey includes 47 direct questions, where participants rate the ease of accessing, interpreting, evaluating, and using health information on a 5-point Likert scale (very easy, easy, difficult, very difficult, and I don't know). The General Health Literacy Index classifies respondents into four categories based on their scores: poor (0–25), problematic (26–33), sufficient (34–42), and outstanding (43–50). In the study, participants scoring 33 or below were categorized as “Inadequate,” while those scoring above 33 were categorized as “Adequate.” The English version of the questionnaire demonstrated internal consistency and reliability, which was assessed by computing Cronbach's alpha, yielding a score of 0.97. Moreover, the tool was previously validated in Debre Tabor, Ethiopia, in a cross-sectional study titled “Information Seeking Behavior About Cancer and Associated Factors Among University Students in Ethiopia” (19). The third section of the questionnaires covers clinical, behavioral, and psychosocial factors, which have been developed, adapted, and refined based on a review of the literature, the conceptual framework, and relevant sources.

Depression, Anxiety, and Stress Scale (DASS-21) was developed by Lovibond and Lovibond's is a validated psychological screening tool designed to assess symptoms of depression, anxiety, and stress. The DASS-21 demonstrates strong reliability, with Cronbach's alpha values of 0.81, 0.89, and 0.78 for its respective subscales. Participants rated their symptoms of depression, anxiety, and stress over the past week using a 7-item scale, with scores ranging from 0 to 3 for each item. These scores were then multiplied by two to obtain the final score. According to the DASS handbook, scores indicating normal levels of depression, anxiety, and stress were categorized as “0” [No], while scores reflecting mild, moderate, severe, or extremely severe levels were categorized as “1” [Yes] (16, 20). Furthermore, the DASS-21 has been utilized multiple times in our country, including in a cross-sectional study conducted in Arsi, Southeast Ethiopia, titled “The Prevalence and Severity of Depression, Anxiety, and Stress among Medical Undergraduate Students of Arsi University and Their Association with Substance Use, Southeast Ethiopia” (21).

Diabetes Knowledge: The Revised Brief Diabetes Knowledge Test (DKT2) was employed to assess diabetes knowledge in adults with type 1 and type 2 diabetes. This test consists of two sections: 14 general knowledge items and nine items related to insulin use. For this study, only the general knowledge items were utilized, with five items excluded due to cultural considerations. Knowledge levels were classified using Bloom's cut-off point, where scores of 80% or higher were deemed indicative of adequate knowledge, while scores below 80% were considered indicative of inadequate knowledge. The internal consistency and reliability were assessed by computing Cronbach's alpha, which resulted in a score of α = 0.843. For this study, only the general knowledge items were utilized, with five items excluded due to cultural considerations, based on previous research conducted in Ethiopia's West Shoa Zone, titled “Factors Associated with Self-Care Practice Among Adult Diabetes Patients in West Shoa Zone, Oromia Regional State, Ethiopia” (20).

Self-Care: The study employed the Revised Summary of Diabetes Self-Care Activities (SDSCA) questionnaire to assess participants' self-care behaviors over the past week. This included adherence to a healthy diet, daily exercise, blood glucose monitoring, foot care, and smoking cessation (21). The study computed mean scores for different domains, including general diet, specific diet, exercise, blood glucose testing, and foot care. Participants were classified based on their overall mean score for diabetic self-care, with those scoring at or above the mean regarded as having good self-care practices, and those scoring below the mean considered to have poor self-care practices. The reliability test evaluated the internal consistency of the tool by calculating Cronbach's alpha, which yielded an overall score of α = 0.680. This method was based on previous studies conducted in Ethiopia, including a study in Gondar titled “Self-Care Practices and Associated Factors Among Patients with Diabetes Mellitus Undergoing Follow-Up at University of Gondar Referral Hospital, Gondar, Northwest Ethiopia” (22).

Self-Rated Health: The study used the Stanford Patient Education Research Center Self-Rated Health Scale to assess participants' self-rated health. The reliability test evaluated the internal consistency of the tool by calculating Cronbach's alpha, which yielded an overall score of α = 0.92 and evaluates health from good to poor (23). Data was collected through face-to-face interviews and document reviews. Two BSc nurses with at least 2 years of clinical experience in the chronic outpatient department were appointed as data collectors, while one nurse was assigned as a supervisor.

### Data quality control

The research used standardized tools, translated from Amharic to English, and retranslated for consistency. Face validity was checked, and data collectors and supervisors received training. a pre-test was conducted a week before the main data collection at Dessie Comprehensive Specialized Hospital, involving 10% of the population. During the pre-test, the internal consistency of the tool was assessed using Cronbach's alpha: Healthcare/Access Information = 0.823, Disease Prevention/Apply Information = 0.914, Health Promotion/Access Information = 0.722, Overall health literacy = 0.92. Then modifications were made after the pre-test. The data collection process began, with data collectors managing redundancy by recording participants' medical records.

### Data processing and data analysis

EpiData software version 4.6.0.4 and SPSS for Windows version 26 were used in the study to analyze data presented in text, tables, and figures. The binary logistic regression model was applied to identify significant variables. Variables with a *p* < 0.25 in the bivariable binary logistic regression were included in the multivariable binary logistic regression model. In this model, variables with a *p* < 0.05 were considered statistically significant. All assumptions for the binary logistic regression model were evaluated and confirmed, Hosmer-Lemeshow test value (0.84). Multicollinearity was assessed using variance inflation factors (VIF) and tolerance, with VIF values below 10 and tolerance values above 0.1 for all predictor variables.

## Result

### Socio-demographic characteristics of the study participants

Out of a total sample of 423 participants, 419 completed the study, resulting in a response rate of 99%. Among the respondents, 230 (55%) were female. The ages of the participants ranged from 18 to 87 years, with a mean (SD) of 49.65 (±18.239) years. Two hundred fifty-two (60%), lived in urban areas. Regarding marital status, more than half (65%), were married. In terms of educational status, ~112 (27%) were illiterate. More than half (79%), were employed. Concerning income, nearly half, 199 (48%), were classified as Below the Poverty Line. Additionally, 254 (61%) of the respondents were enrolled in community-based health insurance (Table 1).

**Table 1:** Socio-demographic characteristics of type 2 diabetic follow-up participants attending WCSH, Woldia, Northeast Ethiopia from April 19 to July 19, 2022 (*n* = 419).

**Variable**	**Category**	**Frequency (*N*)**	**Percentage (%)**
Gender	Male	189	45
	Female	230	55
Residency	Urban	252	60
	Rural	167	40
Educational status	Illiterate	112	27
	Literate	307	73
Marital status	Single	146	35
	Married	273	65
Occupational status	Un-employed	88	21
	Employed	331	79
Monthly income	Below poverty line	199	48
	Above poverty line	220	52
Age in years^a^	18–35	110	26
	36–50	104	25
	51–65	106	25
	≥66	99	24
CBHI enrolment	Yes	254	61
	No	165	39
Distance from health institution in kilometer	≥5	204	49
	<5	215	51

a Age category was adopted from a research article (a study done in Kigali; 46).

CBHI, Community-Based Health Insurance.

### Clinical characteristics

The median (IQR) duration of diabetes among participants was 4 years. Regarding chronic diabetic-related complications, less than half, 88 (21%), had developed complications, with 88 (21%) experiencing only one such complication. The most common complication was diabetic-related hypertension, affecting 59 (14%) of participants. Of the total participants, 284 (68%) were using insulin only. Additionally, 98 (23%) had developed co-morbid conditions, with hypertension being the most prevalent, affecting 96 (23%). Among all respondents, 143 (34%) owned a personal glucometer, 17 (4.1%) were members of a diabetic association, and 215 (51%) lived within a 5-kilometer distance from a health institution. More than half, 240 (57%), rated their health as good. Regarding self-care practices, 239 (57%) had poor self-care, and 235 (56%) had inadequate knowledge about diabetes (Table 2).

**Table 2:** Clinical characteristics and diabetic knowledge of type 2 diabetic follow-up participants attending WCSH, Woldia, Northeast Ethiopia from April 19 to July 19, 2022 (*n* = 419).

**Variable**	**Category**	**Frequency (*N*)**	**Percentage (%)**
Chronic complication of DM	Yes	88	21
	No	331	79
Number of complications	One complications	88	21
	Two complications	0	0
	More than two complications	0	0
Type of complications	Diabetic nephropathy	6	1
	Diabetic retinopathy	12	3
	Diabetic neuropathy	11	3
	Diabetic foot ulcer	0	0
	Diabetic heart disease	0	0
	Diabetic hypertension	59	14
Treatment regimens	Oral anti-diabetic Medication only	112	26
	Insulin only	284	68
	Insulin and oral anti-diabetic medication	20	5
	Only following the dietary plan as recommended	3	1
Co-morbidity	Yes	98	23
	No	321	77
Type of comorbidity	Heart related disease	0	0
	Kidney disease	6	1
	Hypertension	96	23
Diabetes duration in years	≥5	179	43
	<5	240	57
Having personal glucometer	Yes	143	34
	No	276	66
Member of diabetic association	Yes	17	4
	No	402	96
Self-rated Health	Poor	179	43
	Good	240	57
Self-care practice	Poor	239	57
	Good	180	43
Diabetic knowledge	Inadequate	235	56
	Adequate	184	44

### Behavioral and psychosocial characteristics

Of the total participants, 173 (41%) reported 148 (35%) experienced depression, more than half of the study participants (54%) experienced anxiety, and 154 (37%) experienced stress. Among the participants, nearly 90 percent of the study participants (377) had never chewed khat, 410 study participants (98%) had never smoked cigarettes, and 289 study participants (69%) had never consumed alcohol (Table 3).

**Table 3:** Behavioral and psychosocial characteristics of type 2 diabetic follow-up participants attending WCSH, Woldia, Northeast Ethiopia from April 19 to July 19, 2022 (*n* = 419).

**Variable**	**Category**	**Frequency (*N*)**	**Percentage (%)**
Depression	Yes	148	35
	No	274	65
Anxiety	Yes	227	54
	No	192	46
Stress	Yes	154	37
	No	265	63
Alcohol drinking	Yes	130	31
	No	289	69
Khat chewing	Yes	42	10
	No	377	90
Cigarette smoking	Yes	9	2
	No	410	98

### Health literacy level

**Figure 1:**
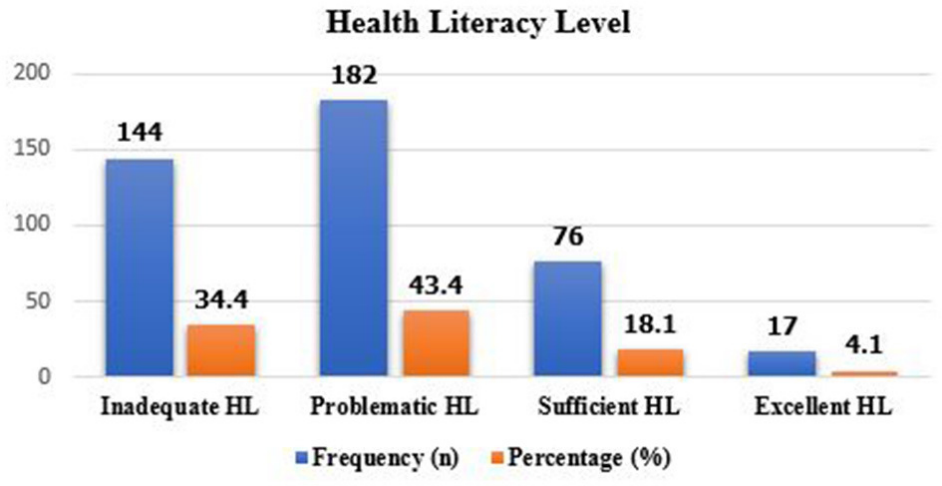
Health literacy level among type 2 diabetic follow-up participants attending WCSH, Woldia, Northeast Ethiopia from april to july 19, 2022 (*n* = 419).

Study participants, had 34.4% inadequate health literacy, 182 (43.3%), had Problematic health literacy, 18.1% had sufficient health literacy and 4.1% had excellent health literacy (Figure 1).

### Magnitude of limited health literacy

**Figure 2:**
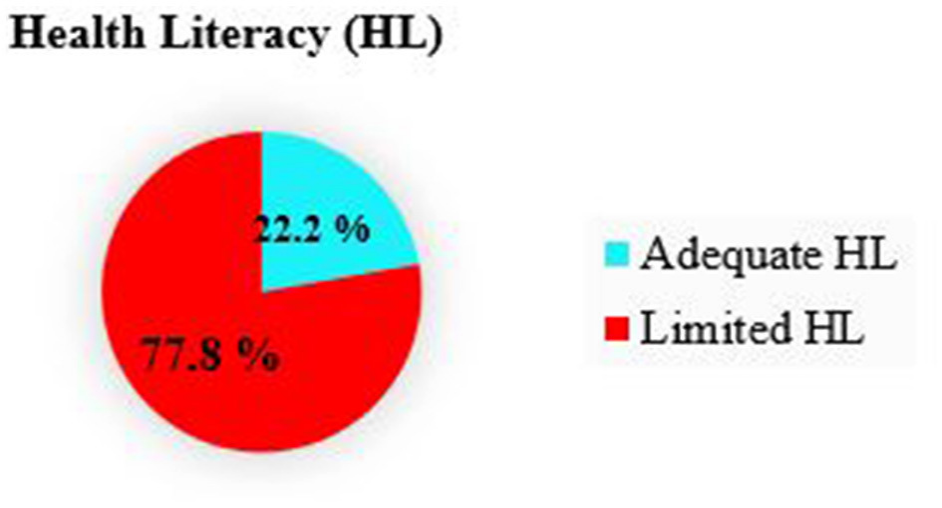
Magnitude of adequate health literacy among type 2 disbetic follow-up participants attending WCHS, Woldia, Northeast Ethiopia from april to july 19, 2022 (*n* = 419).

The Mean (SD) General Health Literacy Index score was 27.8602 (±6.71287). The overall magnitude of adequate health literacy was 22.2 (95% CI 18.4–26.3) whereas, limited health literacy was 77.8% (95% CI 73.8–81.9; Figure 2).

### Factors associated with limited health literacy

The variables included in the bivariable binary logistic regression analysis and subsequently entered into the multivariable binary logistic regression analysis with a *p* < 0.25 were age, gender, residency, educational status, income, depression, and anxiety. The multivariable binary logistic regression analysis, conducted using the backward selection method, identified age (18–35) years, age (36–50) years, gender, educational status, depression, and anxiety were significantly associated with adequate health literacy (LHL).

Respondents aged (18–35) years were 14 times more likely to have adequate health literacy as compared to those aged (>=66) years [(AOR =14, 95% CI = 3.86–50.77)].

Respondents aged (36–50) years are 15.38 times more likely to have adequate health literacy as compared with those aged (>=66) years [(AOR = 15.38, 95% CI = 4.23–55.9)].

Being male was 2.945 times more likely to have adequate health literacy as compared to female respondents [(AOR = 2.945, 95% CI = 1.570–5.526)].

Respondents who have not experienced depression symptoms were 2.673 times more likely to have adequate health literacy as compared to respondents who have experienced depression symptoms [(AOR = 2.673, 95% CI = 1.308–5.463)].

Respondents who have not experienced anxiety symptoms were 2 times more likely to have adequate health literacy as compared to respondents who have experienced anxiety symptoms [(AOR = 2.001, 95% CI = 1.011–3.960)].

Being literate was 4.120 times more likely to have adequate health literacy as compared to being illiterate [(AOR = 4.120, 95% CI = 1.397–12.146; Table 4)].

**Table 4:** Multivariable binary logistic regression on factors associated with adequate health literacy among type 2 diabetic follow-up participants attending WCSH, Woldia, Northeast Ethiopia from April 19 to July 19, 2022 (*n* = 419).

**Variable categories**		**Health literacy**		**COR (95% CI)**	**AOR (95% CI)**	***P*-value**
		**Adequate** = **1** ***n***	**Limited** = **0** ***n***			
Sex	Male	63	126	3.3 (2–5.43)	2.945 (1.570–5.526)	0.001
	Female	30	200	1	1	
Residency	Urban	70	182	2.4 (0.9–3.5)	1.914 (0.960–3.817)	0.065
	Rural	23	144	1	1	
Depression	No	44	104	1.92 (1.2–3)	2.673 (1.308–5.463)	0.007
	Yes	49	222	1	1	
Anxiety	No	37	155	0.73 (0.46–1.17)	2.001 (1.011–3.960)	0.047
	Yes	56	171	1	1	
Income	Above poverty line	43	177	0.72 (0.46–1.15)	0.555 (0.285–1.079)	0.083
	Below poverty line	50	149	1	1	
Educational status	Literate	88	219	8.6 (3.4–21.8)	4.120 (1.397–12.146)	0.010
	Illiterate	5	107	1	1	
Age (18–35) year		38	72	5.28 (2.4–11.63)	14 (3.86–50.77)	0.0001
Age (36–50) year		33	71	4.65 (2–10.3)	15.38 (4.23–55.9)	0.0001
Age (51–65)		13	93	1.4 (0.57–3.4)	3.078 (0.821–11.540)	0.096
Age (>=66) year		9	90	1	1	

^*^Statistically significant (*p* < 0.05); ^**^statistically highly significant (*p* < 0.01).

AOR, adjusted odds ratio; CI, confidence interval; COR, crude odds ratio.

## Discussion

The magnitude of adequate health literacy among type 2 diabetic follow-up patients in this study was 22.2% (95% CI: 18.4–26.3). According to the findings of this study, type 2 diabetic patients did not receive sufficient health information from healthcare providers. This highlights significant obstacles to diabetic self-care across various health domains, including environmental, social, psychological, physical, and spiritual aspects. One study carried out in Iran indicated that the best way to improve the health and quality of life of people with type 2 diabetes is to create a health literacy promotion program that aims to improve self-care practices (5). Another study condcucted in Southern California on Resident Physician Empathy and Health Literacy Communications associated with diabetic control approved that Patients' understanding of the self-management required for effective diabetes control may be improved through compassionate communication from resident physicians that aligns with their health literacy levels (24).

This study's findings were lower compared to studies conducted at Jimma Comprehensive Specialized Hospital of Ethiopia (53%; 10) and the University Of Gondar Comprehensive Specialized Hospital Of Ethiopia (56.5%; 31). The differences may be due to the findings of this study used 47-items health literacy tool, participants with diabetes for <5 years, a more representative sample size, and the majority experiencing anxiety. Similarly, the findings of this study was lower than Switzerland, Rwanda, Malaysia, “Atlanta and Chicago”, Brazil, Georgina, Iran, Kuwait, and Zabol with magnitudes of adequate health literacy were (33%), (44.7%), (34.7%), (86%), (73.3%), (67%), (36.3%), (35.5%), and (28.7%) (26), (27), (40), (28), (29), (30), (31), (32), and (34) respectively.

The differences observed in the findings of this study can be attributed to several factors. About 40% of participants lived in rural areas, which limited their access to information from media sources like television and radio. Additionally, around 27% of participants were illiterate, and ~48% had incomes below the poverty line. Furthermore, about 66% did not have a glucometer to monitor their blood glucose at home, and 56% lacked adequate knowledge about type 2 diabetes mellitus. As a result, many participants developed chronic complications, such as diabetic retinopathy, diabetic neuropathy, and hypertension. Additionally, around 54% experienced anxiety and about 31% were addicted to alcohol, highlighting significant health challenges compared to other studies. The assessment and decision-making components of health literacy were shown to be responsible for about 25% of the self-care practices in a similar study that was carried out in Tehran on the prediction of self-care behaviors among patients with type 2 diabetes (7).

Being male was significantly associated with adequate health literacy compared to being female. This study's findings align with similar studies conducted in Iran and Saudi Arabia (31, 35). Gender disparities in health literacy related to non-communicable diseases may arise from historically greater educational access for men, more proactive health information-seeking behaviors, and better access to health services. Consequently, men tend to engage more in preventive health behaviors.

Study participants who have not experienced depression symptoms and anxiety showed a positive relationship with adequate health literacy compared to those who have experienced depression symptoms and anxiety. This study finding was consistent with studies conducted in Switzerland and Saudi Arabia (26, 35, 36).

The relationship may be influenced by several factors. Participants without depression or anxiety tend to have better attention, memory, and engagement in health management, enabling them to seek and understand health information effectively. In contrast, those with depression may struggle, resulting in inadequate health literacy about type 2 diabetes. This highlights the need for targeted interventions to improve health literacy for individuals with depression and anxiety.

Literate study participants are strongly associated with adequate health literacy as compared to illiterate ones. This study was concurrent with studies conducted in Turkey, Rio de Janeiro, Brazil, India, Iran, Norway, Jimma Comprehensive Specialized Hospital of Ethiopia, Kuwait, Malaysia, and Saudi Arabia (15, 36, 41, 43, 44, 46, 47, 51) (43–45). Literate individuals have better access to health information, make informed decisions, and enhance critical thinking, which helps them interpret health materials and communicate effectively with providers. This promotes awareness of health issues and preventive measures. Therefore, healthcare providers and organizations should prioritize community engagement in health education to prevent type 2 diabetes and its complications during clinical follow-ups and early screenings.

Study participants whose ages are (18–35) and (36–50) years were strongly associated with adequate health literacy as compared with those whose ages >=66 years. This study aligned with studies conducted in Brazil, Iran, Kuwait, Rio de Janeiro, and Saudi Arabia (29, 31, 32, 35). Young and middle-aged adults are generally more proficient with digital tools for accessing health information, have better cognitive function, and are more familiar with current health guidelines than older adults. Therefore, healthcare providers should prioritize older adults with type 2 diabetes during clinical follow-ups, as their health significantly impacts overall outcomes.

### Limitations of the study

The study's limitations include methodological weaknesses, such as cross-sectional nature, potential social desirability bias and recall bias, which may limit its generalizability to the general population and may lead to biased results.

## Conclusion

The overall magnitude of adequate health literacy results was lower as comparable to other studies. Being male, ages (18–35), (36–50) years, not experiencing depression symptoms, not experiencing anxiety, and being literate were significantly associated with adequate health literacy.

## Data Availability

The original contributions presented in the study are included in the article/supplementary material, further inquiries can be directed to the corresponding author.
